# Breeding blueberries for a changing global environment: a review

**DOI:** 10.3389/fpls.2015.00782

**Published:** 2015-09-30

**Authors:** Gustavo A. Lobos, James F. Hancock

**Affiliations:** ^1^Faculty of Agricultural Sciences, Plant Breeding and Phenomic Center, Universidad de TalcaTalca, Chile; ^2^Department of Horticulture, Michigan State UniversityEast Lansing, MI, USA

**Keywords:** Vaccinium, drought, heat, UV, phenotype, highbush, MAB, phenomics

## Abstract

Today, blueberries are recognized worldwide as one of the foremost health foods, becoming one of the crops with the highest productive and commercial projections. Over the last 100 years, the geographical area where highbush blueberries are grown has extended dramatically into hotter and drier environments. The expansion of highbush blueberry growing into warmer regions will be challenged in the future by increases in average global temperature and extreme fluctuations in temperature and rainfall patterns. Considerable genetic variability exists within the blueberry gene pool that breeders can use to meet these challenges, but traditional selection techniques can be slow and inefficient and the precise adaptations of genotypes often remain hidden. Marker assisted breeding (MAB) and phenomics could aid greatly in identifying those individuals carrying adventitious traits, increasing selection efficiency and shortening the rate of cultivar release. While phenomics have begun to be used in the breeding of grain crops in the last 10 years, their use in fruit breeding programs it is almost non-existent.

## Introduction

Over the last 100 years, the geographical area where highbush blueberries are grown has expanded dramatically (Retamales and Hancock, 2012). The northern highbush blueberry (NHB) is native to the eastern and mid-western portions of the USA, where winters are very cold, summers are moderate and chilling hours are high (Table 1). The Industry was first established in New Jersey (1910), but within a few decades had expanded to North Carolina (1920), Michigan (1930), and the Pacific Northwest (1940). From there it leapfrogged to Europe (1970s), New Zealand/Australia (1980s), central Chile (1980s), and most recently China (2000s).

**Table 1:** **Climates of major global highbush and rabbiteye blueberry production regions (adapted from Retamales and Hancock, 2012)**.

**Region**	**State**	**City**	**Rainfall (mm)**		**Temperature (**^**°**^**C)**				**Frost-free days**	**Chilling hours (7°C)**
			**Annual**	**Summer**	**Mid-summer high**	**Mid-summer low**	**Mid-winter high**	**Mid-winter low**		
North America (Atlantic)	N. Carolina	Wilmington	1378	478	33.0	21.0	14.0	1.5	246	500–800
North America (Northeastern)	New Jersey	Hammonton	1097	284	30.0	19.0	5.0	−4.5	182	1000+
North America (Midwestern)	Michigan	Holland	1021	294	27.0	14.0	−2.0	−10.0	156	1000+
North America (Southeastern)	Florida, north	Gainesville	1234	495	33.5	22.5	22.0	10.0	285	150–350
	Florida, central	Orlando	1228	528	33.0	21.5	19.0	6.0	315	400–500
	Georgia	Alma	1248	432	33.5	22.0	17.0	5.0	250	450–600
	Mississippi	Poplarville	1606	414	33.5	22.0	15.5	3.5	256	450–600
North America (Northwestern)	British Columbia	Vancouver	1201	134	21.5	13.0	6.0	0.5	170	1000+
	Oregon	Corvallis	1168	92	28.0	13.5	9.0	2.0	190	1000+
	Washington	Vancouver	1267	71	25.0	12.0	7.5	0.0	177	1000+
North America (Southwestern)	California	Bakersfield	163	5	36.0	21.0	13.5	4.0	277	450–550
	Mexico	Guadalajara	927	676	32.4	16.8	26.5	10.2	365	0
Africa	South Africa	Cape Town	515	52	26.1	15.7	17.5	7.0		400–600
Asia	China	Dalian	632	405	26.1	20.7	−0.9	−7.7		1000+
	Japan	Tokyo	1465	481	30.8	24.2	9.8	2.1		1000+
Europe	Poland	Warsaw	520	203	23.6	12.9	0.4	−4.8		1000+
	Germany	Hamburg	773	224	22.1	12.7	3.5	−1.4		1000+
	France	Bordeaux	986	179	22.6	15.2	10.0	2.8		1000+
	Spain	Huelva	490	16	29.6	21.4	16.1	7.0		200–400
	Netherlands	Amsterdam	778	194	21.8	12.5	5.4	0.2		1000+
	Italy	Venice	810	154	27.5	17.8	5.8	−0.9		1000+
Pacific Rim	Australia	Coffs Harbor	1704	570	27.0	19.0	19.1	7.0		400–500
		Melbourne	665	154	24.8	14.5	13.4	5.9		800+
	New Zealand	Auckland	1135	246	25	14	16	7		800+
South America	Chile, north-central	Santiago	311	3	29.7	13.0	14.9	3.9		800+
	Chile, south-central	Osorno	1383	160	23.8	8.6	11.3	3.8		800+
	Argentina	Buenos Aires	1215	348	30.4	20.4	14.9	7.4		300–400

The expansions into the Pacific Northwest, Mexico, and Chile were into climates with much less severe winters, while the introductions into China were into much colder regions. Cultivars developed in Michigan and New Jersey have generally thrived in the milder climates of the Pacific Northwest, but many of them suffer from the high irradiance in Chile and the cold of China.

Southern highbush blueberry (SHB) types were originally developed in the 1980s by incorporating genes from native species from the southern US to reduce the chilling requirement of NHB. SHB were first established in Florida and Georgia (1980s) and then moved to north central Chile (1980), Argentina and Spain (1990), California (2000) and most recently Mexico, Peru and Ecuador (2010s).

The introductions of SHB into California, north Central Chile, and Spain were into hotter and dryer climates than those in Florida and Georgia (Table 1), and in southern Chile with much higher UV levels (Huovinen et al., 2006). The expansions into Mexico and Ecuador were from low to moderate chill conditions to regions with few to no hours under 7°C. In general, the cultivars that have done well in Florida and Georgia have also performed well in the hotter, drier production regions of California, central Chile, and Spain. However, only a few low chill cultivars have performed well in Mexico and Peru, and many of them suffer under the high UV levels of Chile.

During the last couple of decades, a constant stream of successful cultivars has been released from a number of breeding programs. These programs have focused on releasing cultivars with reduced chilling hours in warmer regions, increased cold hardiness in colder regions, and higher performance under high pH, temperature and radiative stress, but there is still much room for improvement. To achieve these goals, blueberry breeders have incorporated genes from many species within the *Vaccinium* genus through inter-specific hybridization (Table 2), which should prove to be a rich genetic pool for further improvements.

**Table 2:** **Genetic composition of some of the cultivated blueberries**.

**Cultivar**	**Specie composition (%)**							
	**VC**	**VA**	**VD**	**Va**	**VT**	**Vc**	**VE**	**Others**
Elliott, Brigitta, Liberty, Aurora, Lateblue, Jersey	100.0							
Duke	96.0	4.0						
Bluecrop	93.6	6.4						
Hannah's Choice	92.2	7.8						
Avonblue	86.7	0.8	5.0	7.5				
Lenoir	85.2	2.3	12.5					
Draper	84.5	6.0	1.6	1.2	0.4			6.3
O'Neal	84.0	10.0	3.0	3.0				
Misty	81.0	1.0	9.0	6.0	3.0			
Ozarkblue	77.3	3.9	11.3	7.5				
Summit	77.3	3.9	11.3	7.5				
Reveille	77.2	4.1	3.1	2.3	0.8			12.5
Sampson	76.6	10.9	12.5					
Magnolia	75.5	5.7	10.0	7.5	1.3			
Legacy	73.4	1.6	25.0					
Star	71.9	7.7	7.2	5.9	1.0			6.3
Camellia	71.8	1.6	19.7	3.8				3.1
Bluetta, Patriot, Sunrise	72.0	28.0						
Carteret	71.5	3.5					25.0	
Millennia	66.5	5.3	1.3	1.9				25.0
Jubilee	56.6	2.7	26.9	7.5				6.3
Emerald	54.4	1.9	13.9	1.5	0.2			28.1
Sierra	50.0	2.0	20.0	15.0		13.0		
Cara's Choice	47.7	2.3	20.0	15.0		15.0		
Sharpblue	43.7	28.8	15.0					12.5
Biloxi	41.8	1.8	32.5	11.3				12.6

*VC, V. corymbosum; VA, V. angustifolium; VD, V. darrowii; Va, V. ashei; VT, V. tenellum; Vc, V. constablei; VE, V. elliottii (Hancock and Siefker, 1982; Ehlenfeldt, 1994; Clark et al., 1996; Hancock et al., 1997; Brevis et al., 2008; Lee et al., 2012; Rowland et al., 2013)*.

In this paper, we review the environmental challenges facing blueberry cultivation due to global warming. We describe the state of the art of blueberry breeding and outline how future varietal development can be enhanced by marker assisted breeding (MAB) and phenomics.

## Environmental challenges to blueberry cultivation

The expansion of highbush blueberry growing into colder and warmer regions will be challenged by the alterations in global temperature and rainfall patterns, both associated with increases in atmospheric CO_2_ concentrations. From the “Industrial Revolution” carbon dioxide has increased in a significant way and will continue to do so. It is estimated that under the most conservative scenario, atmospheric CO_2_ concentrations at the end of the century will be at least double to the pre-industrial era, increasing by 35% from year 2005 (IPCC, 2007b). As atmospheric concentrations of greenhouse gases rise due to the human activity, worldwide climatic patterns are being greatly altered (United Nations, 2010). The Intergovernmental Panel on Climate Change (IPCC), reports that during the past 150 years, global mean temperatures raised 0.045°C per decade, but in the last 25 years have increased almost four times (0.177°C) (IPCC, 2007a). Two separate analyses done recently by NASA (National Aeronautics and Space Administration) and NOAA (National Oceanic and Atmospheric Administration) have concluded that 2014 was the warmest year since 1880 (NASA, 2015). It is expected that during the next century global temperatures will be increased by an additional 1.1–6.4°C (Jin et al., 2011).

The increases in temperature are associated with extreme variations in weather patterns, resulting in severe droughts, unusually heavy rains and atypically hot temperatures (Allen and Ingram, 2002). Since the 1970s, the frequency of warm nights and days is increasing dramatically (IPCC, 2007a). For example, in the main blueberry production area in Chile (approximately between 35 and 38° Latitude S), precipitation diminished around 25% during the 20th Century, and it is estimated that there will be a further reduction of 5–15% over the next 30 years (Meza et al., 2003; Santibañez and Santibañez, 2007, 2008; United Nations, 2010). These dramatic changes led Friend (2010) to suggest that “Quantifying and explaining the current global distribution of plant production, and predicting its future responses to climate change and increasing atmospheric CO_2_, are therefore major scientific objectives.” High temperatures and drought can significantly reduce the productivity and the quality of the harvested organ (Moretti et al., 2010), restricting the areas (latitudes and soils) where economically important species can be grown.

The activity and development of humanity has not only increased atmospheric CO_2_ levels but also levels of chlorofluorocarbons from aerosols, refrigerators, and other equipment that conditions the air. These compounds destroy the ozone layer, which selectively absorbs ultraviolet light. Ozone absorbs 100% of UV-C, prevents the passage of UV-B (near 90%) but does not affect the UV-A transmission (de Gruijl and van der Leun, 2000). In the southern (35–60°) and northern (35–60°) hemisphere, the annual mean ozone quantities during 2006–2009 were lower than between 1964 and 1980 (6 and 3.5%, respectively) (WMO, 2011).

The average UV erythemal irradiance, which indicates potential biological damage to human skin from solar ultraviolet radiation, has steadily risen as the amount of ozone has decreased (WMO, 2011). Compared with the 1970s, surface erythemal UV radiation has increased 7% in winter-spring and 4% in summer-fall in the northern hemisphere mid-latitudes, 6% year-round in the mid-southern hemisphere latitudes, and 22% in the Antarctic and Arctic in the spring (Madronich et al., 1998). In the summertime, erythemal UV irradiance in the southern hemisphere is up to 40% higher than values in the northern hemisphere (Madronich et al., 1998). If the Montreal Protocol is followed, it is possible that UV values will return to 1980 levels by the middle of this century, but this is dependent on multifaceted global cooperation (Kazantzidis et al., 2010; McKenzie et al., 2011).

## Implications of climate change on blueberry breeding

The aspect of global warming that most needs attention from blueberry breeders is the dramatic seasonal fluctuations now occurring in rainfall and temperature patterns. Cultivars well adapted to “average conditions,” often do not have sufficient plasticity to perform well under the range of conditions now being faced. For example, an unusually warm spring in Michigan in 2012 lead to very early floral development, and as a result, when temperatures returned to normal later in the spring, a high percentage of flowers were damaged by frost. An unusually hot summer in the Pacific Northwest in 2012, resulted in the fruit of most cultivars being too soft for extended storage. This was followed by an unusually cold winter in 2013–2014, where high percentages of the floral buds were heavily damaged. In Chile, falls and winters are becoming progressively milder in many areas, causing some cultivars to bloom out of season (O'Neal, Snowchaser and Misty, among others).

To maintain and extend the geographic range where blueberries are grown, breeders will need to be much more cognizant of the potential range of environments that the cultivars will face. They will need to take care not to release cultivars that are narrowly adapted to average conditions. Among the environmental challenges faced by blueberry breeders are:

### Winter cold

The range of the highbush blueberry has been limited by extreme winter cold. Cold hardiness is a complex interaction between rate of acclimation (development of freezing tolerance) and deacclimation (loss of developed freezing tolerance), as well as degree of mid-winter tolerance. This is extremely important since unseasonably warm midwinter spells can trigger a premature deacclimation, exposing the bush to freeze damage (Arora and Rowland, 2011).

In general, northern highbush cultivars survive much colder mid-winter temperatures than southern highbush ones, although considerable variability exists within groups and among *Vaccinium* species (Hancock et al., 1997; Ehlenfeldt et al., 2003, 2006, 2007; Dhanaraj et al., 2004; Rowland et al., 2004; Ehlenfeldt and Rowland, 2006; Hanson et al., 2007). In full dormancy, northern highbush genotypes have been found to range in tolerance from −20 to −30°C. Few southern highbush have been evaluated, although “Legacy” tolerates temperatures to −17°C and “Ozarkblue” to −26°C. US 245, an inter-species hybrid of US 75 (“Bluecrop” × *V*. *darrowii* “Fla 4B”) × “Bluecrop,” is tolerant to at least −24°C.

To date, the primary approach to developing more cold tolerant blueberries has been to hybridize lowbush with highbush to produce “half-high” types (Trehane, 2004; Hancock et al., 2008a). However, the shorter stature of the half-highs and the fact they become covered and protected with snow may be the primary basis of their increased tolerance (El-Shiekh et al., 1996). Due to the lack of formal comparisons of flower bud tolerance to winter cold in highbush, lowbush and half-highs, it is unknown how much more cold tolerant highbush can be made through introgression. It would be productive to determine if several other wild species carry useful genes for cold hardiness including *V. boreale, V. constablaei*, and *V. myrtilloides* (Galletta and Ballington, 1996; Ehlenfeldt and Rowland, 2006). Ehlenfeldt et al. (2007) showed that when *V. ashei* was hybridized with *V. constablaei*, cold hardiness was positively associated with the percentage of *V. constablaei* genes.

Little formal genetic analysis of cold tolerance of tetraploid blueberry has been performed. Arora et al. (2000) found in diploid populations that the cold hardiness data fit a simple additive-dominance model of gene action, with the additive effects being greater than the dominance ones. During cold acclimation, specific genes are expressed in floral buds that increase cold tolerance (Naik et al., 2007). Arora et al. (1997a), working with “Bluecrop,” “Tifblue,” and “Gulfcoast,” found a close relationship between floral bud dehydrin concentration and the level of cold hardiness. Similar results were found by Rowland et al. (2004) and Dhanaraj et al. (2005). This suggests that dehydrin concentration might be a way to predict the cold hardiness of selections in a breeding program.

It is important to note that studies of cold hardiness under field or artificial conditions can lead to different conclusions. When “Bluecrop” (NHB) and “Tifblue” (Rabbiteye blueberry—RE) flower buds were assessed in the field, LT_50_ (maximum level of cold-hardiness) were close to −27 and -25°C (respectively), whereas the same cultivars in cold room conditions (4°C) reached maximums around −24 and −17°C, respectively (Arora et al., 1997b; Arora and Rowland, 2011). There were almost twice as many “Bluecrop” genes expressed in the cold room than in the field, suggesting that many of the genes induced in the cold room were responding to low temperature (specifically 4°C) and were not contributing to freezing tolerance *per se*. In contrast, more “Tifblue” genes were expressed in the field than under the controlled conditions. This suggests that there is a strong genotype × environment interaction associated with cold tolerance and any screen designed to select cold hardy genotypes, must be conducted under field conditions or under realistic controlled protocols (Arora and Rowland, 2011).

It may be possible to determine when a plant is approaching full dormancy by measuring the expression of the β-amylase gene. Lee et al. (2012) showed in the NHB “Jersey” and the SHB “Sharpblue” that there was an abrupt reduction in starch in shoots in the middle of cold acclimation, which was associated with an increase in the expression of the β-amylase gene. This change was positively correlated with the total amount of soluble solids in the wood, which likely served as osmoprotectants able to reduce the freezing point. Inter-species differences in the level of expression of β-amylase genes in northern and southern highbush were described by Rowland et al. (2008).

### Spring and fall frost

Freezing damage to developing flowers in the spring is a major problem in most blueberry production regions, with both NHB and SHB. It is a rare year when at least a fraction of the flower buds is not damaged. Rate of deaclimation likely plays a role in early spring flower bud tolerance. Ehlenfeldt (2003) found the northern highbush “Duke” deacclimated the fastest in a mixed group of 12 cultivars, while the southern highbush “Magnolia,” the northern highbush × rabbiteye pentaploid hybrid “Pearl River,” the rabbiteye × *V*. *constablaei* “Little Giant” and the half-highs “Northcountry” and “Northsky” were the slowest. Northern highbush “Bluecrop” and “Weymouth,” southern highbush “Legacy” and “Ozarkblue” were intermediate. While there is evidence of considerable variability, no formal genetic studies have been done on deaclimation rates.

Identifying late bloom or slower deacclimating genotypes will be useful for breeding spring-frost tolerant cultivars (Rowland et al., 2005). Because of the chances of frosts and the direct relation between the stage of floral development and the relative bud hardiness, those cultivars with late bloom dates tend to suffer less frost damage than those flowering earlier (Spiers, 1976; Hancock et al., 1987; Patten et al., 1991; Lin and Pliszka, 2003). When Hancock et al. (1987) assessed flower bud injury in 18 highbush blueberry cultivars after two spring frosts in Michigan, they found significant differences in proportion of brown ovaries among cultivars, ranging from 25 to 94%. Most of the variation was associated with stage of bud development.

Bloom date is strongly correlated with ripening date, but early ripening cultivars have been developed that have later than average flowering dates such as the NHB “Duke,” “Huron” and “Spartan,” and the SHB “Santa Fe” and “Star.” Bloom date, ripening interval and harvest dates are highly heritable in blueberry populations (Lyrene, 1985; Hancock et al., 1991) with strong genotype by environmental interactions (Finn et al., 2003). Finn and Luby (1986) found additive genetic variation was more important than non-additive effects for date of 50% bloom, 50% ripe fruit and for length of fruit development interval in populations from hybrids between *V. angustifolium* and *V*. *corymbosum*. Where spring frosts are a problem, breeders can focus on developing cultivars with late bloom dates and where earliness is premium, selection will need to be made on ripening interval as well.

Flower buds also can be damaged by rapid freezes in the fall. The flower buds of SHB cultivars are generally considered to acclimate more slowly in the fall than those of NHB ones, and as a result are more subject to late fall freezes; however, few formal screens of germplasm have been conducted on this characteristic (Rowland et al., 2005, 2013; Hanson et al., 2007). Leaf retention in the fall does not appear to be a good predictor of rate of DA, as Hanson et al. (2007) found that “Ozarkblue” and US 245 retain their leaves until the very late fall, but they are just as hardy as the mid-season standard “Bluecrop.” Bittenbender and Howell (1975) also found no correlation between flower bud hardiness and fall leaf retention.

There are not many studies that have evaluated the effect of the spring frost on open flowers, and most of them have been done on RE (Spiers, 1976; Gupton, 1983; NeSmith et al., 1999). Among RE cultivars, “Southland” proved to be more frost-tolerant than “Delite,” “Woodard,” “Climax,” and “Tifblue” (Gupton, 1983). Nevertheless, interspecific crosses with RE would not be recommended to increase bud tolerance to frost since, at similar stages of floral bud development, RE tend to be more sensitive than NHB and SHB (Patten et al., 1991). Rowland et al. (2013), studying the sensitiveness of five northern highbush blueberries cultivars (“Bluecrop,” “Elliott,” “Hannah's Choice,” “Murphy,” and “Weymouth”) to frost damage of open flowers, concluded that “Hannah's Choice” and “Murphy” were the most tolerant whereas “Bluecrop” was the most susceptible. Among the cultivars analyzed by Rowland et al. (2013), female parts from “Elliott” (styles), “Hannah's Choice” (styles and exterior ovaries) and “Murphy” (styles) were more frost-tolerant than those structures of “Bluecrop,” and the male organs from “Murphy” (filaments and anthers) were more frost-tolerant than “Bluecrop.” These differences need to be studied in other cultivars and exploited for breeding.

### Chilling requirement

Expanding the range of adaptation of the NHB by reducing its chilling requirement has been a major breeding goal over the last 50 years (Hancock et al., 2008a). This was largely accomplished by incorporating genes from the southern diploid species *V. darrowii* into *V. corymbosum* via unreduced gametes, although hybridizations with native southern *V. corymbosum* and *V. ashei* also played a role. Cultivars with an almost a continuous range of chilling requirements (hours below 7°C) are now available from 0 to 1000 h.

Most SHB are grown in areas with 250–600 chilling hours each winter. SHB cultivars vary widely in their performance without any chilling hours. Surprisingly, one of the best adapted cultivars to this system is “Biloxi,” which requires 500 chilling hours (< 7°C) in Mississippi, where it was developed. The response to chilling is clearly a complex interaction and many factors play a role including sensitivity to temperature shifts, floral development time, response to photoperiodic change and temperature thresholds.

The genetics of the chilling requirement has not been formally determined, although segregation patterns suggest that it is largely quantitatively inherited with the low chilling requirement showing some dominance. The precise temperature necessary to break dormancy has not been determined, but Mainland et al. (1977) and Spiers (1976) have proposed that the chilling requirement of highbush blueberries is at least partially satisfied by temperatures below 1.4 and above 12.4°C. It is possible that blueberry genotypes vary in the threshold temperatures that are required to break dormancy, although this has not been documented. Southern highbush cultivars with complex ancestry may be particularly variable in their temperature thresholds.

### Heat and drought tolerance

When heat stress is present in blueberries, the quick response needed to supply the atmospheric demand, puts the plant and its fruit at permanent threat (Chen et al., 2012). High summer temperatures, such as in the subtropical southeast China or the dry Mediterranean north of Chile, impact on the productivity of highbush blueberries across much of their range (Darnell, 2000; Chen et al., 2012). It is thought that SHB are more tolerant of high temperatures than NHB, but both types commonly experience summer temperatures in the field that have negative impacts on CO_2_ assimilation rates and fruit quality. In general, optimal temperatures have been shown to vary between 20 and 25°C (Davies and Flore, 1986).

No formal studies have been conducted on the genetics of photosynthetic heat tolerance in blueberry, but genetic variation has been documented. Moon et al. (1987b) evaluated the optimum temperature for photosynthesis in different highbush cultivars, determining ranges of 18–6°C for Jersey and 14–22°C for Bluecrop. A temperature of 30°C has been shown to reduce photosynthesis in NHB cultivars by 22–51% (Hancock et al., 1992); the authors reported that “Jersey,” “Elliott,” and “Rubel” showed a decrease in photosynthesis between 22 and 27% whereas for “Spartan,” “Bluejay,” and “Patriot” it was between 41 and 51%. Trehane (2004) describes “Ozarkblue” and “Jubilee” as varieties that perform well in hot summers. Chen et al. (2012) found that at high temperatures, up to 40–45°C, a number of photosynthetic parameters were damaged in “Brigitta,” but they stayed largely intact in “Sharpblue,” and “Duke”; “Misty” performed in the middle. In this study, at high temperatures, there were increases in hydrogen peroxide, super oxide radical and F_0_ (minimum fluorescence in the dark-adapted state), while Fv/Fm (maximum photochemical quantum yield of photosystem II) and ÔPS II (quantum efficiency of PSII photochemistry) decreased.

Southern highbush cultivars may have obtained higher photosynthetic heat tolerance from *Vaccinium darrowii* (Lyrene, 2002). Moon et al. (1987a) found that CO_2_ assimilation (*A*) in Fla 4B of *V. darrowii* was similar at 20 and 30°C, while *A* in the pure northern highbush “Bluecrop” dropped by almost 30% across this same range. Transpiration rates were also much lower in Fla 4B than “Bluecrop.” This difference was found to be heritable, with a tetraploid F_1_ hybrid actually having higher *A* than the two parents (Moon et al., 1987b; Hancock et al., 1992). The selection, Fla 4B has been used to generate many of the important southern highbush cultivars including “Biloxi,” “Emerald,” “Legacy,” and “Star” (Lyrene and Sherman, 2000; Draper and Hancock, 2003). There may also be additional sources of heat tolerance in the native southern species *V. tenellum, V. myrsinites, V. pallidum, V. ashei, V. elliottii, V. stamineum, Vaccinium arboreum*, southern diploids and tetraploids of *V. corymbosum* (Luby et al., 1991).

It would seem likely that the photosynthetic heat tolerance of both NHB and SHB types can be increased by crossing the most heat tolerant genotypes, since there is considerable genetic variability for this trait both within and among blueberry species.

High temperatures also negatively impact fruit quality and storage life of highbush blueberries. Temperatures higher than 32°C during the maturation of the fruit can give rise to smaller, soft fruits and with waxes that have greater susceptibility of being lost by means of the rubbing (by leaves or during the harvest) (Mainland, 1989).

Blueberries have a relatively inefficient water conducting systems, characterized by the lack of root hairs (Gough, 1994). Root anatomy and architecture should be a key trait, but unfortunately it is almost unexplored, e.g., *V. arboreum* is drought tolerant specie because it has deep tap roots in contrast to the spreading, shallow root systems of highbush blueberry. Hence, drought tolerance of highbush blueberries might also be enhanced by using species material in breeding.

In his screens of wild species material, Erb et al. (1988a,b) found *V. elliottii, V. darrowii*, and *V. ashei* to be the most drought tolerant species and this characteristic was transmitted to hybrid progeny. Moon et al. (1987a) found transpiration rates and leaf conductance (*gs*) to water vapor to be much lower in *V. darrowii* than “Bluecrop” at high temperature. This suggests that *V. darrowii* may have higher drought tolerance through decreased stomatal opening and subsequent restriction of water loss.

Other sources of drought tolerance likely include the native species *Vaccinium stamineum* and *V. arboretum* (Hancock et al., 2008a). *V. stamineum* is the most drought tolerant species in the southeastern U.S.A., but hybrids derived with species in section Cyanococcus have not been vigorous (Ballington, 1980; Lyrene, 2006). The use of *V. arboreum* appears to be more promising, as this species can be crossed with *V. darrowii* to produce vigorous hybrids, and these hybrids can be used as a bridge to tetraploid SHB types (Lyrene, 1991; Brooks and Lyrene, 1998; Olmstead et al., 2013).

In response to water deficit, plants stop shoot growth affecting their final height and diameter (Mingeau et al., 2001). Bluecrop is one of the cultivars which has been studied most, proving to be highly sensitive to water deficit, showing a rapid stomatal closure, and reduced gas exchange (Cameron et al., 1989; Rho et al., 2012), berry size and yields (Améglio et al., 2000). When “Bluecrop” was subjected to severe hydric restriction, a reduction in the yield (31–49%) was observed (Perrier et al., 2000). Similar results were found for other highbush cultivars like Rancocas (Lee et al., 2006) and Jersey (Cameron et al., 1989). Rho et al. (2012) also found that along with the reduction in gas exchange found in “Bluecrop” under water deficit (−1.9 MPa), an increment in the electron transport rate (ETR) occurs, indicating photorespiration is also affected.

When Estrada et al. (2015) studied how SHB, RE, and NHB responded to drought conditions, with or without heat stress, they found that under each stress SHB and RE had a better photoprotection capacity, while the NHB showed increments in its photochemical capacity. When both stresses were present, just NHB “Liberty” and “Elliott” had increased ETR_max_ (maximum electron transport rate), the coefficient of photochemical fluorescence quenching (qP and qL) and the effective photochemical quantum yield of photosystem II [Y(II)]. This suggests these two cultivars should be considered as parents if reduction of photo-oxidative damage is required. This tool could be used to screen genotypes much faster than other classic measurements, such as gas exchange rate, chlorophyll content, stem water potential, etc., making the monitoring of stressed plants more efficient (Ralph and Gademann, 2005; Estrada et al., 2015).

The priority of this trait for the breeder varies depending on location and irrigation availability. It should be mentioned that currently most blueberries are cultivated under irrigation or with irrigation supplementation.

### High UV light

Ozone depletion itself, is not a major contributor to global warming, but increases in UV irradiance have large, direct impacts on plant productivity (Boesgaard et al., 2012). In some latitudes, plants will not only have to deal with extreme photosynthetic active radiation and heat, but also with high UV radiation. Is not unusual to observe damaged plants in commercial fields (Yáñez et al., 2009), as well as among breeding families, in central Chile with reddened and curled leaves and what appear to be localized burns on fruit. Shading nets have been shown to enhance productivity of blueberries in central Chile, but the direct influence of UV light has not be investigated (Retamales et al., 2008; Lobos et al., 2009, 2012, 2013, 2015).

Kakani et al. (2003), reviewing 129 studies of the effect of UV-B on 35 crops, reported that higher levels of UV-B (most affected by the ozone depletion) were associated with vegetative and reproductive morphology alterations, decreases in chlorophyll content and photosynthesis, and chlorotic or necrotic patches on leaves or fruit. Little formal work has been done on the effect of UV light on northern highbush blueberries, and most of what has been done has focused on postharvest improvement through short treatments of UV-B on harvested fruit (Perkins-Veazie et al., 2008; Eichholz et al., 2011).

The damaging effects of high UV light have been documented in other *Vaccinium* species. Albert et al. (2008) and Boesgaard et al. (2012) found a reduction of net photosynthesis in *V. uliginosum* throughout the season and damage to photosystem II (PS II) through the diminution of the fluorescence measured as Fv/Fm. Kossuth and Biggs (1978) tested the effects of 15, 24, and 44 units of UV-B on the rabbiteye blueberry “Woodard” and found that the higher doses reduced fruit growth and surface bloom, and under high doses of UV-B the fruit skin actually appeared burned.

Among the mechanisms that might be selected to improve UV tolerance in blueberries is the ability of the leaf surface to reflect part of the incident radiation (Semerdjieva et al., 2003a,b). The thickness of the epidermis of the leaf and the concentration of absorbent compounds could also be improved to counteract the damaging effects of UV radiation (Batschauer, 1998; Boesgaard et al., 2012). Increases in levels of phenols (flavonoids and hidroxamic acid), could also help counter the degradative effects of high UV-B on DNA (Rozema et al., 1997; Ruhland et al., 2005). Defense responses to UV-B have not been evaluated in highbush blueberry; however, Semerdjieva et al. (2003a,b) noted in three other species (*V. myrtillus* L., *V. vitis-idaea* L., and *V. uliginosum* L.) in the north of Sweden, that there were noticeable differences in quantity of phenolic compounds. In all species, high UV-B led to an increase in phenolic compounds, but some genotypes responded more than others, and the plants with the highest flavonoid content had the least UV-B damage.

## New frontiers for the breeding of blueberries

The breeding of highbush blueberries can be a long and tedious process. Traditional approaches take from 10 to 20 years from the original cross to cultivar release and often the precise adaptive range of a cultivar is not known until farmers have grown it for a number of years. Two relatively new techniques called “MAB” and “Phenomics” could greatly facilitate blueberry breeding. MAB would aid in the selection of those individuals most likely to carry adventitious traits and Phenomics would allow for much more easy, fast and precise characterization of the superior types. It is possible that individuals could be selected for their adaptability to variable environmental conditions with MAB even though they were not exposed to those conditions in the field.

### Marker assisted breeding (MAB)

All breeding programs revolve around identifying the optimal traits for a cultivar. Most blueberry breeding programs utilize traditional approaches to identify desirable types, such as walking along rows of crosses in the field or doing simple laboratory assays on fruit quality and disease resistance. However, in many other crops, MAB is used to facilitate and speed up the release of new cultivars (Cabrera-Bosquet et al., 2012; Araus and Cairns, 2014).

MAB is based on DNA diagnostic tests that can identify potential parents and progeny carrying desirable traits. This process allows selection to be moved all the way back to conception in the breeder's minds, helping them to only make crosses that create desirable trait combinations in offspring, increasing the efficiency of the entire process. It also permits selection to be moved from the field to the greenhouse, so that only seedlings predicted to be superior are planted in the field for further evaluation. In addition, MAB allows for the assessment of traits that are difficult to predict in the field such as chilling hour requirement or heat tolerance.

To use MAB to broaden the environmental range where highbush blueberries can be grown, it will be necessary to find genetic variability associated with expanded adaptations. The rich germplasm diversity currently being used by blueberry breeders (Table 2) is likely to contain useful genes. The major stumbling block to using MAB will be the collection of precise data on the adaptations of potential breeding parents. Evaluations of genotypes in the field will require that the extreme conditions occur when the plants are in the field (Arora and Rowland, 2011). Care will need to be taken to evaluate genotypes in appropriate environments and in many cases controlled experiments will need to be undertaken. Most likely a genotype will need to be evaluated in multiple environments to obtain an accurate representation of its adaptations. This will often be tedious and time consuming, but once markers are found that are tightly linked to the genes regulating adaptations of interest, future screening will be greatly facilitated through MAB. As we discuss below, field screening could be greatly streamlined using phenomic techniques involving spectrometry and thermography.

The first genetic maps of blueberries are beginning to emerge that will set the groundwork for MAB. Rowland's group at the USDA-ARS (Genetics of Fruit and Vegetable Improvement Laboratory, Beltsville, MD, USA) developed the first blueberry map using a diploid population segregating for chilling requirement (Rowland and Levi, 1994). Their population was a cross between an inter-specific hybrid (*V. darrowii* × *V. corymbosum*) and another clone of *V. corymbosum*. They have continued to periodically add markers to this map and at last report had 265 markers mapped to 12 linkage groups. They have used this map to identify quantitative trait loci for cold tolerance and chilling requirement (Rowland et al., 2014). Allan Brown (North Carolina State University, NC, USA) and Eric Jackson (General Mills, Crop Bioscience, MN, USA) have led teams that sequenced the genome of a *V. darrowii* × *V. corymbosum* hybrid and used this information to generate a more dense chromosome map with 1200 markers.

A major research initiative has been undertaken by Lisa J. Rowland, Nahla Bassil (USDA-ARS, National Clonal Repository, Corvallis, OR, USA), Julie Graham and Susan McCallum (The James Hutton Institute, Dundee, UK) and Jim Olmstead (University of Florida, Gainesville, FL, USA) to develop a linkage map of the tetraploid cross “Jewel” (SHB) × “Draper” (NHB) (Rowland et al., 2012). Replicated progeny of that cross were planted at five locations across the USA and data was collected on a wide array of traits including fruit quality, developmental rates, chilling hour requirements and growth patterns. A QTL analysis is currently being conducted to search for markers for these traits.

The first few thousand expressed sequence tags (ESTs) have been generated and made publicly available for the Ericaceae family, about 5000 from blueberry and about 1200 from rhododendron (Rowland et al., 2010, 2014; Die and Rowland, 2013) (http://bioinformatics.towson.edu/BBGD/). These ESTs from blueberry and rhododendron were generated as parts of projects focused on cold acclimation research, and the ESTs are from non-acclimated and cold acclimated flower bud libraries, in the case of blueberry (Dhanaraj et al., 2004, 2007), and from non-acclimated and cold acclimated leaf libraries, in the case of rhododendron (Wei et al., 2005). Another ~16,000 ESTs have been generated from blueberry fruit by the New Zealand Institute for Plant and Food Research Ltd. (formerly HortResearch), but they are not publicly available.

### Phenomics

The success of breeding programs is reflected in the number of individuals released at the end of the selection process (Hancock et al., 2008b). In order to be successful, breeders must generate thousands of hybrids annually and evaluate them for a number of years. What ultimately is selected is dependent on local environmental conditions (Araus and Cairns, 2014).

Because of the large number of genotypes that need to be evaluated, deep phenotypic characterizations of the material often becomes impractical due to the time and costs involved (White et al., 2012; Kipp et al., 2014). For this reason, conventional breeding generally focuses on different visual characteristics (e.g., fruit color, cluster tightness, disease resistance, growth habit, flowering, and ripeness dates) and a few that require measurements of average complexity (e.g., yield, soluble solids, firmness).

**Figure 1:**
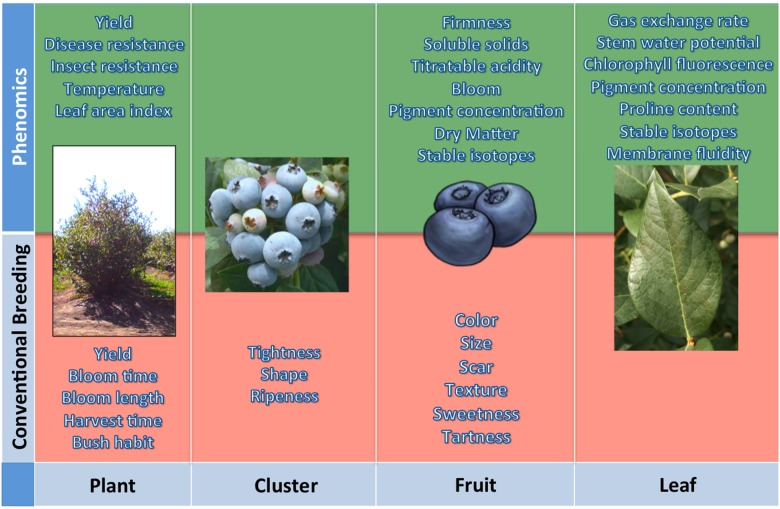
**The traits commonly evaluated by a plant breeder are highlighted in red**. Others that can be estimated by phenomics are highlighted in green—mostly of these are valuable morpho-physiological and physico-chemical traits that most breeders are not able to consider.

To effectively develop cultivars well adapted to fluctuations in environmental stresses, blueberry breeders will have to evaluate a number of morpho-physiological and physico-chemical traits that they are not used to considering (Figure 1). The only reasonable way to fulfill all these needs is through acquisition of high-dimensional phenotypic data (high-throughput field phenotyping) or “Phenomics” (Houle et al., 2010). Nowadays, there are a number of remote sensing devices, techniques and analysis, mostly non-destructive, which have proven very helpful in the characterization of the phenotype (Furbank and Tester, 2011; White et al., 2012; Araus and Cairns, 2014).

Among the remote sensing technologies with the greatest potential for use in phenomics are spectrometry and thermography. Spectroradiometers are widely used to measure plant reflectance (R), whose spectral signature (graphic characterization of reflected segment of wavelengths) is closely associated with the absorption at certain wavelengths that are linked to specific characters or plant conditions (Araus and Cairns, 2014). Thermography uses plant temperature as an efficient tool for the study of the spatial and temporal heterogeneity of plant water status and how it responds to the environment.

**Figure 2:**
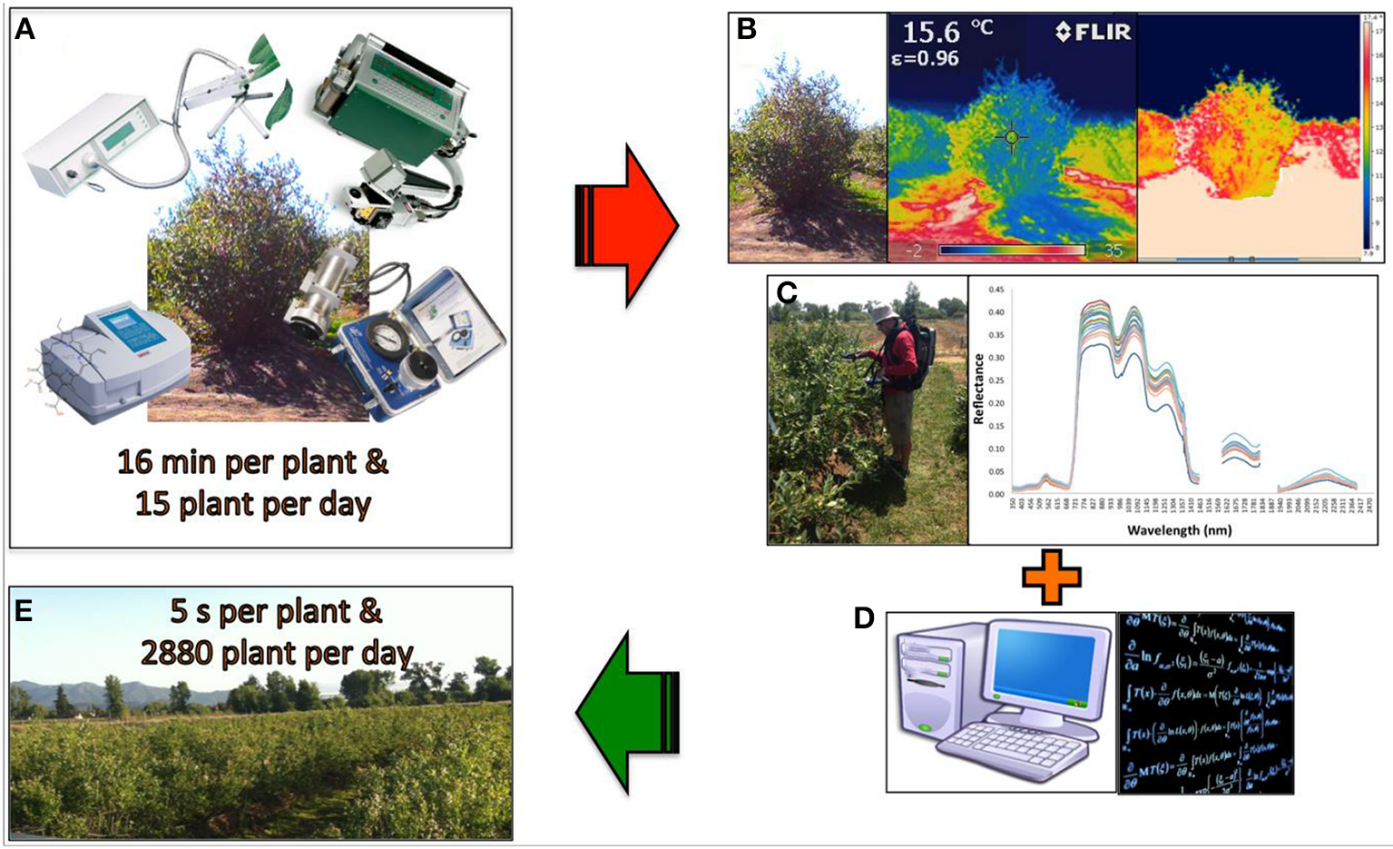
**When drought or heat stress needs to be assessed, the evaluation window per day is reduced to 4 h**. A basic physiological plant evaluation (gas exchange rate, stem water potential, chlorophyll fluorescence, and pigment concentration) **(A)** takes about 16 min per plant, representing a characterization of a maximum 15 genotypes per day. When thermography **(B)** and spectrometry **(C)** is considered, linear and non-linear modeling **(D)** can streamline the evaluation of plant status, further increasing the number of genotypes that can be evaluated in a breeding population per day **(E)**.

While these techniques are not new, their use was expanded tremendously during mid 1980s and is now being widely used in plant ecophysiology and postharvest studies (estimation of yield, nutritional content in leaves, gas exchange rate, fruit quality, biotic and abiotic stress, etc.) (Garriga et al., 2014; Lobos et al., 2014). Measurements that before usually took months, weeks, or days can now be accomplished in hours or even minutes for a large number of genotypes (Figure 2).

In spectrometry, reflectance data is used to generate “Spectral Reflectance indices” (SRI). Initially SRIs were simple relationships between wavelengths or spectral bands. The first SRI was the “Simple Ratio,” calculated as the ratio of the near infrared (NIR) to the visible (VIS) (SR = R_NIR_/R_VIS_), and the “Normalized Difference Vegetation Index” [NDVI = (R_NIR_-R_VIS_)/(R_NIR_+R_VIS_)]. Since then, and incorporating specific wavelengths, SRIs have been used in different species to estimate green biomass and leaf area index (Tucker and Sellers, 1986), plant water status (Peñuelas et al., 1993), radiation use efficiency (Peñuelas et al., 1995), water content in leaves (Sims and Gamon, 2003), photosynthetic capacity and efficiency (Inoue et al., 2008), micro and macro nutrients in leaves (Basayigit and Senol, 2009), yield and carbon isotope discrimination (Lobos et al., 2014) among many others.

Since the 1960s, plant temperature has been widely used as an indicator of water status (Tanner, 1963). Initially, temperature measurements were performed by thermocouples in contact with the leaves. Later, the development of infrared sensors allowed faster measurements of leaves or canopies. With thermal imaging it is possible to detect biotic or abiotic pre-symptomatic responses, providing a powerful tool to evaluate a high number of samples in only a few minutes (Costa et al., 2013; Araus and Cairns, 2014). The development of cheaper devices has made this approach available to farmers and breeders. Thermometry analysis has been fine-tuned over time and the different parts of the image (soil, air, leaves, stems, branches, etc.) can now be isolated, allowing for the evaluation of specific tissues, organs or individuals (Costa et al., 2013; Araus and Cairns, 2014).

To date, spectrometry and thermography have been used to only a limited extent on blueberries. There are a few studies where the antioxidant content in NHB was evaluated using spectrometry (total phenols, total flavonoids, total anthocyanins, and ascorbic acid) (Sinelli et al., 2008; Bai et al., 2014). The ideal “Brigitta” harvest date was determined in the field using reflectance data (Beghi et al., 2009), and a blueberry ripeness index (BRI) has been developed (Beghi et al., 2013). Guidetti et al. (2009) used a portable spectroradiometer Vis-NIR to accurately estimate soluble solids, firmness and functional compounds (anthocyanins, flavonoids, polyphenols, and ascorbic acid) in fresh and homogenized fruit samples of “Brigitta” and “Duke.” Other work on blueberries focused on monitoring osmo and air dehydration processes (Sinelli et al., 2011), SHB cultivar identification (Yang and Lee, 2011; Yang et al., 2012), and the recognition of foreign materials (leaves and stems) among frozen blueberries (Tsuta et al., 2006; Sugiyama et al., 2010). In the wild lowbush blueberry (*V. angustifolium*) reflectance data has also been used for detection of internal larvae fruit infestation (Peshlov et al., 2009), *in situ* levels of foliar nitrogen (Bourguignon, 2006; Maqbool et al., 2012) and to evaluate vegetative (leaf area index) and reproductive (flower number, fruit set, and berry yield) parameters (Percival et al., 2012). Most recently, hyperspectral imaging has been used to predict soluble solids content and firmness in NHB fruit (Leiva-Valenzuela et al., 2013, 2014), to identify damaged fruit (Leiva-Valenzuela et al., 2012; Leiva-Valenzuela and Aguilera, 2013), to classify blueberry fruit growth stages (Yang et al., 2012, 2014), and as a tool for early detection of leaf rust in blueberries (Ahlawat et al., 2011). Escobar-Opazo (2015) found that in blueberry some of the physiological parameters were significantly correlated with reflectance data (e.g., ETR_max_ and chlorophyll a/b > 0.90; *A* and *gs* > 0.65).

During the last decade, spectrometry and thermography have begun to be used in the breeding of grain crops, but their use in fruit breeding programs is almost non-existent. Even in the grain crops, their use has been limited to the evaluation of small numbers of genotypes, in general < 20. SRI will need to be replaced by more complex bio-mathematical models, to fully provide breeders with the solid and reliable information they need for plant selection. When this happens, the efficiency of selection should dramatically improve, and well adapted genotypes will be released at a faster rate. The future challenge will be to develop techniques that can screen a large number of genotypes simultaneously (hundreds or thousands) (Figure 2).

## Concluding remarks and future perspectives

Considerable genetic variability exists in the highbush blueberry germplasm base that can be used by breeders to meet the environmental challenges associated with climate change. There has already been extensive range expansion of blueberries into hotter and drier environments. There is no reason to believe that there is not additional genetic variability that can be deployed to further enhance cold acclimation, heat, high UV, and drought tolerance of blueberries. Perhaps the greatest challenge associated with climate change and blueberry range expansion will be the development of blueberry cultivars that can resist extremes in environmental variability. Ongoing research to develop DNA diagnostic markers for key physiological tolerances will aid greatly in the breeding of these stress resistant types. To generate robust markers for MAB, it will be necessary to have precise phenotypic characterizations, making phenomics a powerful tool that could aid greatly in identifying the superior types.

### Conflict of interest statement

The authors declare that the research was conducted in the absence of any commercial or financial relationships that could be construed as a potential conflict of interest.
